# Rigorous Neural Network Simulations: A Model Substantiation Methodology for Increasing the Correctness of Simulation Results in the Absence of Experimental Validation Data

**DOI:** 10.3389/fninf.2018.00081

**Published:** 2018-11-26

**Authors:** Guido Trensch, Robin Gutzen, Inga Blundell, Michael Denker, Abigail Morrison

**Affiliations:** ^1^Simulation Lab Neuroscience, Jülich Supercomputing Centre, Institute for Advanced Simulation, JARA Jülich Research Centre, Jülich, Germany; ^2^Institute of Neuroscience and Medicine (INM-6) and Institute for Advanced Simulation (IAS-6) and JARA Institute Brain Structure-Function Relationships (INM-10) Jülich Research Centre, Jülich, Germany; ^3^Faculty of Psychology, Institute of Cognitive Neuroscience Ruhr-University Bochum, Bochum, Germany

**Keywords:** reproducibility, verification and validation, model validation, SpiNNaker, fixed-point numeric, spiking network models

## Abstract

The reproduction and replication of scientific results is an indispensable aspect of good scientific practice, enabling previous studies to be built upon and increasing our level of confidence in them. However, reproducibility and replicability are not sufficient: an incorrect result will be accurately reproduced if the same incorrect methods are used. For the field of simulations of complex neural networks, the causes of incorrect results vary from insufficient model implementations and data analysis methods, deficiencies in workmanship (e.g., simulation planning, setup, and execution) to errors induced by hardware constraints (e.g., limitations in numerical precision). In order to build credibility, methods such as verification and validation have been developed, but they are not yet well-established in the field of neural network modeling and simulation, partly due to ambiguity concerning the terminology. In this manuscript, we propose a terminology for model verification and validation in the field of neural network modeling and simulation. We outline a rigorous workflow derived from model verification and validation methodologies for increasing model credibility when it is not possible to validate against experimental data. We compare a published minimal spiking network model capable of exhibiting the development of polychronous groups, to its reproduction on the SpiNNaker neuromorphic system, where we consider the dynamics of several selected network states. As a result, by following a formalized process, we show that numerical accuracy is critically important, and even small deviations in the dynamics of individual neurons are expressed in the dynamics at network level.

## 1. Introduction

Even for domain experts, it is often difficult to judge the correctness of the results derived from a neural network simulation. The factors that determine the correctness of the simulation outcome are manifold and often beyond the control of the modeler. It is therefore of great importance to develop formalized processes and methods, i.e., a systematic approach, to build credibility. This applies not only to the modeling, implementation, and simulation tasks performed in a particular study, but also to their reproduction in a different setting. Although appropriate methods exist, such as verification and validation methodologies, they are not yet well-established in the field of neural network modeling and simulation. One reason may lie in the rapid rate of development of new neuron and synapse models, impeding the development of common verification and validation methods, another is likely to be that the field has yet to absorb knowledge of these methodologies from fields in which they are common practice. This latter point is exacerbated by partly contradicting terminology around these areas.

In this study, we propose a reasonable adaptation of the existing terminology for model verification and validation and apply it to the field of neural network modeling and simulation. We introduce the concept of *model verification and substantiation* and apply it to the issue of reproducibility on a worked example. Specifically, we quantitatively compare a minimal spiking network model capable of exhibiting the development of polychronous groups, as described in Izhikevich (2006), to its reproduction on the SpiNNaker (a contraction of Spiking Neural Network Architecture) neuromorphic system (Furber et al., 2013). The Izhikevich (2006) study is highly cited as an account of how spike patterns emerge from network dynamics, and contains a number of non-standard features in its conceptual and implementational choices that make it a particularly illustrative example for the verification process. The choice of a network reproduction implemented on SpiNNaker as a target for comparison is motivated by the fact that SpiNNaker is subject to rather different constraints from typical simulation platforms, in particular the restriction to fixed-point arithmetic, and so demonstrates interestingly different verification problems. With this process we demonstrate the value of software engineering methodologies, such as refactoring, for verification tasks.

Moreover, this study contributes to a question that is intensively debated in the neuromorphic community: how do hardware constraints on numerical precision affect individual neuron dynamics and, thus, the results obtained from a neural network simulation? We compare the neuronal and network dynamics between the original and the SpiNNaker implementation, and our results show that numerical accuracy is critically important; even small deviations in the dynamics of individual neurons are expressed in the dynamics at network level.

This study arose within a collaboration using the same initial study to examine different aspects of rigor and reproducibility in spiking neural network simulations, which we describe briefly here to motivate the scope of the current study. Firstly, a frequent source of errors in a neural network simulation is unsuitable choices of numerics for solving the system of ordinary differential equations underlying the selected neuron model. In section 3.4.2 we focus on the issues of time step and data type; the question of which solver to use is addressed in Blundell et al. (2018b), who present a stand-alone toolbox to analyze the system of equations and automatically select an appropriate solver for it. Secondly, a key aspect of our study is the reproduction of the network described in Izhikevich (2006) on SpiNNaker, as described in sections 3.1.2 and 3.4.1. The difficulties of creating such a reproduction are comprehensively examined by Pauli et al. (2018). Their investigation of the features of source code that support or diminish the reproducibility of a network model is based on reproducing the Izhikevich (2006) study in the NEST simulator (Gewaltig and Diesmann, 2007). In addition to developing a checklist for authors and reviewers of network models, they demonstrate that the reported results are extremely sensitive to implementation details. Finally, in order to determine whether two simulations are producing results of acceptable similarity, we employ a statistical analysis of spiking activity. This is summarized in section 3.2.2; the complete description and derivation of this analysis can be found in our companion paper (Gutzen et al., 2018).

## 2. Terminology

### 2.1. Reproducibility and replicability

Reproducibility and replicability are indispensable aspects of good scientific practice. Unfortunately, the terms are defined in incompatible ways across and even within fields.

In psychology, for example, *reproducibility* may mean completely re-doing an experiment, whereas *replicability* refers to independent studies that yield similar results (Patil et al., 2016). For computational experiments, where the outcome is usually deterministic^1^, *reproducibility* is understood as obtaining the same results by a different experimental setup conducted by a different team (Association for Computing Machinery, 2016; see also Plesser, 2018). Although attempts were made to help resolve the ambiguity in the terminology by explicitly labeling the terms or by attempting to inventory the terminology across disciplines (Barba, 2018), the problem persists. Plesser (2018) gives a brief history of this confusion.

In this study, we follow the definitions suggested by the *Association for Computing Machinery* (Association for Computing Machinery, 2016):

**Replicability** *(Different team, same experimental setup) The measurement can be obtained with stated precision by a different team using the same measurement procedure, the same measuring system, under the same operating conditions, in the same or a different location on multiple trials. For computational experiments, this means that an independent group can obtain the same result using the authors own artifacts*.
**Reproducibility** *(Different team, different experimental setup) The measurement can be obtained with stated precision by a different team, a different measuring system, in a different location on multiple trials. For computational experiments, this means that an independent group can obtain the same result using artifacts which they develop completely independently*.

To be more specific about the terminology of reproducibility, in this study we aim for *results reproducibility* (Goodman et al., 2016; see also Plesser, 2018).

**Results reproducibility** *Obtaining the same results from the conduct of an independent study whose procedures are as closely matched to the original experiment as possible*.

### 2.2. Model verification and validation

The critical question for all modeling tasks is whether the model provides a sufficiently accurate representation of the system being studied. Evaluating the results of a modeling effort is a non-trivial exercise which requires a rigorous validation process.

The term *validation*, or more generally *verification and validation* also require a precise definition, as they have different meanings in different contexts. In software engineering, for example, verification and validation is the objective assessment of products and processes throughout the life cycle. Its purpose is to help the development organization build quality into the system (Bourque and Fairley, 2014). With respect to the development of computerized models, verification and validation are processes that accumulate evidence of a model's correctness or accuracy for a specific scenario (Thacker et al., 2004).

**Figure 1:**
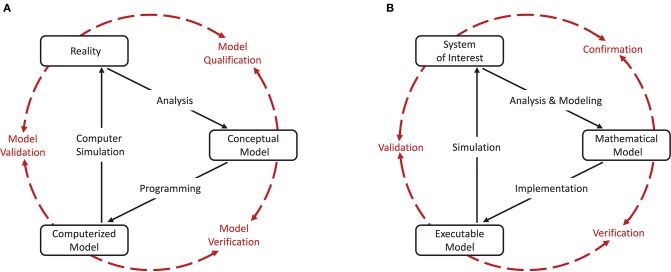
Interrelationship of the basic elements for modeling and simulation. In order to be able to apply the terminology, introduced by Schlesinger et al. (1979) for modeling and simulation processes **(A)**, to numerical models for neural network simulations, a less generic terminology is more expedient. We propose the terminology shown in **(B)** which we have adapted slightly from Thacker et al. (2004). While Thacker et al. (2004) uses the terms *reality of interest, conceptual model*, and *computerized model*, we prefer the terms *system of interest, mathematical model*, and *executable model* as they better express the underlying intent. The model distinguishes between modeling and simulation activities (black solid arrows), and assessment activities (red dashed arrows).

As a cornerstone for establishing credibility of computer simulations, the Society for Computer Simulation (SCS) formulated a standard set of terminology intended to facilitate effective communication between model builders and model users (Schlesinger et al., 1979). This early definition is very general and often does not do justice to a particular modeling domain. Therefore, domain specific adaptations to the terminology can be found, but having fundamentally the same meanings. For the field of neural network modeling and simulation we propose the terminology shown in Figure 1B, amended from Thacker et al. (2004). While Thacker et al. (2004) uses the terms *reality of interest, conceptual model*, and *computerized model*, we prefer the terms *system of interest, mathematical model*, and *executable model*. The terms are more explicit and better express the underlying intent. In particular, due to the empirical challenges of neurobiology, spiking neural network models are often not based on a specific biological network that could be considered “reality” and from which ground truth behavior can be recorded, in contrast to, for example, the air flow around a wing. The term “system of interest” recognizes that the process of verification and validation can also be applied to systems without concrete physical counterparts.

The essence of the introduced terminology is the division of the modeling process into three major elements as illustrated in Figures 1A,B.

Reality or **system of interest** is an “*entity, situation, or system which has been selected for analysis*.” The conceptual or **mathematical model** is defined as a “*verbal description, equations, governing relationships, or natural laws that purport to describe reality or the system of interest”* and can be understood as the precise description of the modeler's intention (Schlesinger et al., 1979). The formulation of the conceptual or mathematical model is derived in a process called **analysis and modeling** and its applicability is motivated in a process termed qualification or **confirmation**. However, the conceptual or mathematical model by itself is not able to simulate the system of interest. By means of applying software engineering and development efforts, it has to be implemented as a computerized or **executable model**.

By separating the understanding of a model into a mathematical and an executable model, this terminology also illustrates the difference between verification and validation.

**Verification** describes the process of ensuring that the mathematical model is appropriately represented by the executable model, and improving this fit.

Model verification is the assessment of a model implementation. Neural network models are mathematical models that are written down in source code as numerical algorithms. Therefore, it is useful to define two indispensable assessment activities:

**Source code verification** tasks confirm that the functionality it implements works as intended.
**Calculation verification** tasks assess the level of error that arises from various sources of error in numerical simulations as well as to identify and remove them (Thacker et al., 2004).

This process mainly involves the quantification and minimization of errors introduced by the performed calculations. Only when the executable model is verified it can be reasonably validated.

The **validation** process evaluates the consistency of the predictive simulation outcome with the system of interest.

The validation process aims at the agreement between experimental data that defines the *ground truth* for the system of interest and the simulation outcomes. This evaluation needs to take into consideration the domain of intended application of the mathematical model as well as its expected level of agreement, since any model is an abstraction of the system of interest and only intended to match to a certain degree and for certain prescribed conditions.

### 2.3. Model verification and substantiation: model assessment in the absence of experimental data

For neural network simulations, the ground truth of the system of interest can be provided by empirical measurements of activity data, for example single unit and multi-unit activity gathered by means of electrophysiological recordings. However, there are a number of reasons why this data may prove inadequate for validation. Firstly, depending on the specification of the system of interest, such data can be scarce. Secondly, even for comparatively accessible areas and assuming perfect preprocessing (e.g., spike sorting), single cell recordings represent a massive undersampling of the network activity. Thirdly, for a large range of computational neuroscientific models, the phenomenon of interest cannot be measured in a biological preparation: for example, any model relying on the plasticity of synapses within a network.

Consequently, for many neuronal network models, the most that the modeler can do with the available experimental data is to check for consistency, rather than validate in the strong sense. Thus, we are left with an incomplete assessment process. However, circumstantial evidence to increase the credibility of a model can be acquired by comparing models and their implementations against each other with respect to consistency (Thacker et al., 2004; Martis, 2006). Such a technique can be meaningful in accumulating evidence of a model's plausibility and correctness even if none of the models is a “*validated model”* that may act as a reliable reference.

To avoid ambiguity with the existing model verification and validation terminology, we propose the term “***substantiation***.”

**Substantiation** describes the process of evaluating and quantifying the level of agreement of two executable models.

**Figure 2:**
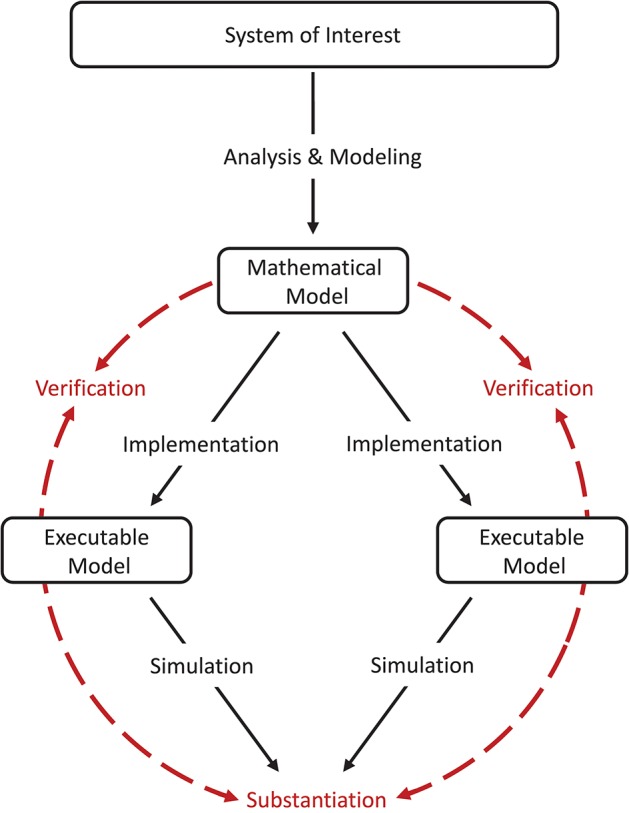
Model verification and substantiation workflow. The workflow shown can be thought of as the combination of two separate model verification and validation processes (Figure 1) without the backward reference to the system of interest, i.e., the validation of the model. In this concept, the consistency of the simulation outcomes of two executable models that share the same system of interest and mathematical model is evaluated, in an assessment activity we term “*substantiation*.” Modeling and simulation activities are indicated by black solid arrows, whereas assessment activities are indicated by red dashed arrows.

*Model verification and substantiation* are then processes that accumulate *circumstantial evidence* of a model's correctness or accuracy by a quantitative comparison of the simulation outcomes from validated or non-validated model implementations. The interrelationship of the modeling, simulation, and assessment activities are shown in Figure 2. To this end, the modeler has to define reasonable acceptance criteria that define the limits within which the process can be executed. In this study, we will demonstrate the usefulness of such an approach.

### 2.4. Application of terminology to neural network modeling and simulation

Applying the given terminology to the domain of neural network modeling and simulations, we will use the terms as follows. *Replication* means using the author's own model, which may consist of the model source code, scripts for network generation and simulation execution as well as additional software components in a particular version (e.g., if a specific simulation software is used). A replication should aim for bit-identicality. Although computers are deterministic, this is not always feasible, for example, if the seed of the pseudorandom number generator has not been recorded, or the generated trajectory of pseudorandom numbers is dependent on the software version or the underlying hardware. Beyond this, replicable models should have the property of delivering exactly the same result in successive simulations on the same hardware. When using random number generators, this entails setting a seed.

A *reproduction* (or specifically, *results reproduction*) is then the re-implementation of the model in a different framework, e.g., expressing a model as a stand-alone script using neural simulation tools, such as NEURON (Hines and Carnevale, 1997), Brian (Goodman and Brette, 2008), NEST (Gewaltig and Diesmann, 2007), or the SpiNNaker neuromorphic system (Furber et al., 2013), and getting statistically the same results.

Applying the terminology defined in this section, one can say: in this study, we replicate a published model and create a reproduction of the model on the SpiNNaker neuromorphic system. In an iterative process of model substantiation, we arrive at the point that both executable models are verified, and in good agreement with one another.

## 3. Model verification and substantiation of the Izhikevich polychronization model: the reproduction of selected network states on spinnaker

### 3.1. Definition of the model verification and substantiation methodology entities

For the purposes of demonstrating a rigorous model verification and substantiation methodology, we define as our *system of interest* the mammalian cortex. A mathematical and executable model of this system was proposed by Izhikevich (2006), who demonstrated that this model exhibits the development of polychronous groups. The mathematical model is described in detail in section 3.1.1, the corresponding executable model, referred to in the following as the *C model*, constitutes one target of the verification and substantiation process illustrated in Figure 2. For the other target, we reproduce the mathematical model on the SpiNNaker neuromorphic system (Furber et al., 2013); the resultant executable model is referred to as the *SpiNNaker model* (see section 3.1.2).

#### 3.1.1. Mathematical model

##### 3.1.1.1. Network topology

**Figure 3:**
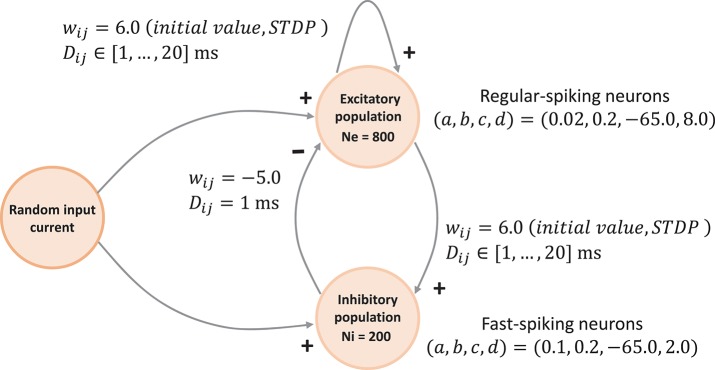
Network architecture. The minimal spiking network exhibiting polychronization as decribed in Izhikevich (2006). The input to the network is a constant current of *I*_ext_ = 20pA into a single neuron, which is randomly selected in each simulation time-step. Please see section 3.1.1 for a detailed description of the mathematical model.

The network connectivity is illustrated in Figure 3. A population of 800 excitatory neurons makes random connections to itself and to a population of 200 inhibitory neurons using a fixed out-degree of 100. Excitatory synaptic connections are initially set to a strength of *w*_*ij*_ = 6.0 and a conduction delay *D*_*ij*_ drawn from a uniform integer distribution such that *D*_*ij*_∈[1, 2, …, 20]ms. The inhibitory population is connected with the same out-degree to the excitatory population only, forming connections with a fixed synaptic strength and delay, *w*_*ij*_ = −5.0, *D*_*ij*_ = 1ms.

##### 3.1.1.2. Component dynamics

Each neuron in the network is described by the simple neuron model presented in Izhikevich (2003), which can reproduce a variety of experimentally observed firing statistics:

(1) $$\begin{array}{r} \overset{{}^{\circ}}{v}=0.04v^{2}+5v+140-u+I \end{array}$$

(2) $$\begin{array}{r} \overset{{}^{\circ}}{u}=a\left(bv-u\right) \end{array}$$

(3) $$\text{if }v\geq 30\text{ m V},\text{ then }\{\begin{array}{l} v\leftarrow c\\ u\leftarrow u+d \end{array}\;.$$

Equations (1)–(3) describe the time evolution of the membrane voltage *v*(*t*) and the threshold dynamic variable *u*(*t*) of a single neuron. For the polychronization model, excitatory neurons are parameterized to show regular-spiking: (*a*, , *b, c, d*) = (0.02, 0.2, −65.0, 8.0), and inhibitory neurons are parameterized to exhibit fast-spiking: (*a, b, c, d*) = (0.1, 0.2, −65.0, 2.0).

The excitatory connections are plastic and evolve according to an additive spike-timing-dependent plasticity (STDP) rule:

(4) $$w\leftarrow \{\begin{array}{l} w+A_{+}\cdot \text{ exp}\left(-\Delta t/\tau_{+}\right)\;:\Delta t\geq 0\\ w-A_{-}\cdot \text{ exp}\left(\Delta t/\tau_{-}\right)\;:\Delta t<0 \end{array}$$

where τ_+_ = τ_−_ = 20 ms, *A*_+_ = 0.1mV, *A*_−_ = 0.12mV, and Δ*t* is the difference in time between the last post-synaptic and pre-synaptic spikes, i.e., positive on occurrence of a post-synaptic spike and negative on occurrence of a pre-synaptic spike. However, the rule has an unusual variant: synaptic weight changes are buffered for one biological second and then the weight matrix is updated for all plastic synapses simultaneously. Thus, synaptic weights are constant for long periods, causing the network dynamics to break down into epochs.

#### 3.1.2. Executable models

##### 3.1.2.1. C model

The original network model and its analysis form a stand-alone application. Several implementations are available for download from the author's website^2^: a MATLAB implementation (*spnet.m*) and two versions of a C/C++ implementation (*spnet.cpp, poly_spnet.cpp*). They differ slightly in algorithms and functionality and thus do not exhibit bit-identical behavior. All implementations use a grid-based simulation paradigm with a resolution of 1ms. Threshold detection according to Equation (3) is performed only at the grid points. For numerical integration of the ODE system consisting of the Equations (1) and (2) a Forward Euler method is used. From the two available versions of the C/C++ implementation we selected the computationally more precise variant *poly_spnet.cpp* that makes use of double precision data types and also implements the analysis, i.e., algorithms for detecting polychronous groups.

##### 3.1.2.2. SpiNNaker model

The SpiNNaker neuromorphic system is a massively parallel multi-core computing system designed to provide a real-time simulation platform for large neural networks (Furber et al., 2013). The largest available system is a half-million core machine^3^. The real-time capability is achieved at an simulation resolution of *h* = 1ms using a grid-based simulation paradigm. This is analog to the integration scheme and simulation paradigm used in the original C model implementation. For our study, we use a SpiNN-3 development board that houses 4 SpiNNaker chips, each containing 18 ARM968 processing cores (Temple, 2011a). For simulation control and cross-development, the SpiNN-3 board must be connected to a host system, which then communicates with the board via Ethernet-based UDP packets (Temple, 2011b). The SpiNNaker software stack (Rowley et al., 2017b) supports the implementation of neural network simulations in PyNN^4^. In addition, it offers several neuron and synapse models as well as a template that enables user to develop custom neuron and synapse models using the event-driven programming model employed by SpiNNaker kernel (Rowley et al., 2017a), available for download from the SpiNNaker repository on GitHub^5^. The SpiNNaker model used in this study was developed from scratch, making use of this template to produce the various Izhikevich neuron model implementations presented in this manuscript.

### 3.2. Definition of the model substantiation assessment

In the absence of specific biological data to define the ground truth for the system of interest, we are left with the simulation outcomes of the two executable models. Here, we consider the dynamics of five selected network states in the C model. The dynamics is assessed by applying statistical analysis methods to the spike train activity data (see section 3.2.2). For an in-depth treatment of the analysis methods used for comparison, see the companion study (Gutzen et al., 2018). Note that we do not use the emergence of polychronous groups or their statistics to define the ground truth, as this turns out to be rather sensitive to details not only of the mathematical model, but also of the implementational choices used to generate the executable model. For a comprehensive investigation of this aspect, see Pauli et al. (2018).

#### 3.2.1. Experimental set-up

**Figure 4:**
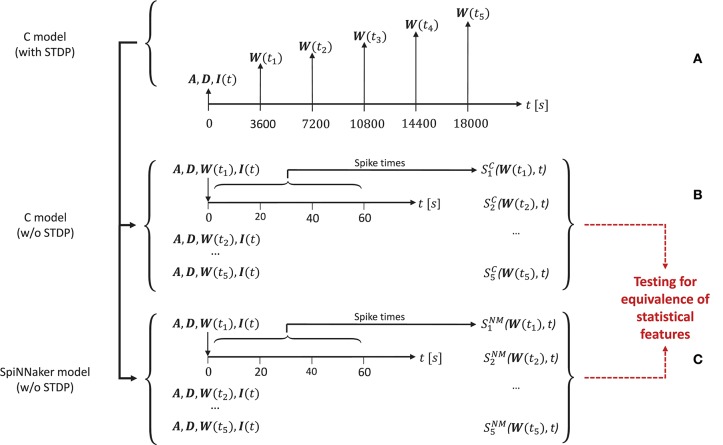
The experimental set-up for the simulations. **(A)** To create the reference data, the C model is executed (with STDP) and the connectivity matrix *A* and delay matrix *D* are saved. Then five times are selected, for which the weight matrix ***W***(*t*_*i*_) is recorded. Along with the input stimulus to the network *I*(*t*), these matrices determine five network states for later comparison. These initial conditions are then set for an implementation of the C model **(B)** and for the SpiNNaker model **(C)**, both without STDP. This results in the network spiking activity recordings $S_{i}^{\text{C}}\left(W\left(t_{i}\right),t\right)$ and $S_{i}^{\text{NM}}\left(W\left(t_{i}\right),t\right)$ for five simulation runs for the C model and the SpiNNaker model, respectively.

In order to generate the network activity data for the comparison tasks carried out in the model substantiation process, we perform the following steps, illustrated in Figure 4.

First, for a given realization (i.e., an implementation and selection of a random seed) for the C model, we execute the model for a duration^6^ of 5 h. During this time we select five times *t*_*i*_, *i* = (1, 2, …, 5) (here: after 1, 2, 3, 4, and 5 h), at which we save the weight matrix ***W***(*t*_*i*_), containing the current strength of each synapse according to the STDP rule described in section 3.1.1. In addition, we save the connectivity matrix ***A***, the delay matrix ***D*** and the first 60 s' worth of the random series of neurons to which an additional stimulus is provided, ***I***(*t*). This procedure is illustrated in Figure 4A.

In a second step, we switch STDP off in the C model. In five consecutive simulation runs, we initialize the network with ***A, D, I***, and the respective ***W***(*t*_*i*_), and record the resultant spiking activity $S_{i}^{\text{C}}\left(W\left(t_{i}\right),t\right)$ over 60 s, as illustrated in Figure 4B. These activity recordings define five dynamic states of the network at different stages of its evolution, constituting the reference data (i.e., fulfilling the role that ground truth data plays in a classical model validation assessment).

Finally, we repeat the second step using the SpiNNaker model (see Figure 4C), resulting in corresponding network activity recordings $S_{i}^{\text{NM}}\left(W\left(t_{i}\right),t\right)$. To perform the model substantiation assessment, the spiking data $S_{i}^{\text{C}}$ and $S_{i}^{\text{NM}}$ are analyzed and compared as described in section 3.2.2.

Note that although the parameters and properties of the polychronization model remain untouched, model implementations do change in successive iterations of the verification and substantiation process as described below; consequently, so do the reference data.

#### 3.2.2. Analysis of network spiking activity

Besides a verification on the level of the dynamics of an individual neuron, we assess the degree of similarity between the different implementations of the Izhikevich polychronization model on the descriptive level of the population dynamics (cf. also, Gutzen et al., 2018). As issues such as the choice of 32/64-bit architecture, floating-point/fixed-point arithmetic, compiler options influencing the evaluation order of expressions or the choice of pseudorandom numbers and the corresponding seed should not be considered part of the mathematical model, it is legitimate and expected that different implementations will not yield an exact spike-by-spike correspondence (but see Pauli et al., 2018 for a counterexample). We therefore resort to testing for equivalence of statistical features extracted from the population dynamics. These tests are conducted in an automated, formal framework that conducts statistical analysis of parallel spike trains using the standardized implementations found in the *Electrophysiology Analysis Toolkit*^7^ (Elephant, RRID:SCR_003833) as its backend. We stress the importance of using a common tool to extract the statistical features for both simulation outcomes in the substantiation procedure in order to prevent distortions in the substantiation outcome due to discrepancies in the implementations of the substantiation procedure itself. In addition, making use of methods provided by such open-source projects greatly contributes to the correctness and replicability of the results.

When choosing the measures by which to compare the network activity, it is essential to assess diverse aspects of the dynamics. Besides widely used standard measures to characterize the statistical features of spike trains or the correlation between pairs of spike trains, this may also include additional measures that reflect more specific features of the network model (e.g., spatio-temporal patterns). Here, we apply tests that compare distributions of three statistical measures extracted from the population dynamics: the average firing rates, the local coefficient of variation as a measure of spike time regularity (Shinomoto et al., 2003), and the pairwise correlation coefficients between all pairs of parallel spike trains (bin width: 2 ms). They can be regarded as forming a hierarchical order and evaluate different aspects of the network dynamics: rates consider the number of observed spikes, whilst ignoring their temporal structure; the local coefficient of variation considers the serial correlations inherent in a spike train, whilst ignoring the relationship between spike trains; the cross correlation considers coordination across neurons.

It should be noted that, as shown later in this study, this conceptual hierarchy does not imply a hierarchy of failure, i.e., a correspondence on the highest level (here: cross correlation) does not automatically imply correspondence of the other measures. Therefore, it is imperative to independently evaluate each statistical property. We evaluate the similarity of the distributions of these measures between simulations using the effect size (Cohen's *d*), i.e., the normalized difference between the means of the distributions (Cohen, 1988). In addition to the substantiation tests selected for the current study, more intricate comparisons can evaluate the correlation structure and dynamical features of the network activity in greater detail, outlined in our companion study (Gutzen et al., 2018).

### 3.3. Definition of the model verification and substantiation workflow

As stated above, model substantiation evaluates the level of agreement between executable models and their implementations, but is not conclusive whether the model itself is correct, i.e., an appropriate description of an underlying biological reality. It is therefore out of scope of this study to evaluate any neuroscientific aspects of the model described in Izhikevich (2006).

**Figure 5:**
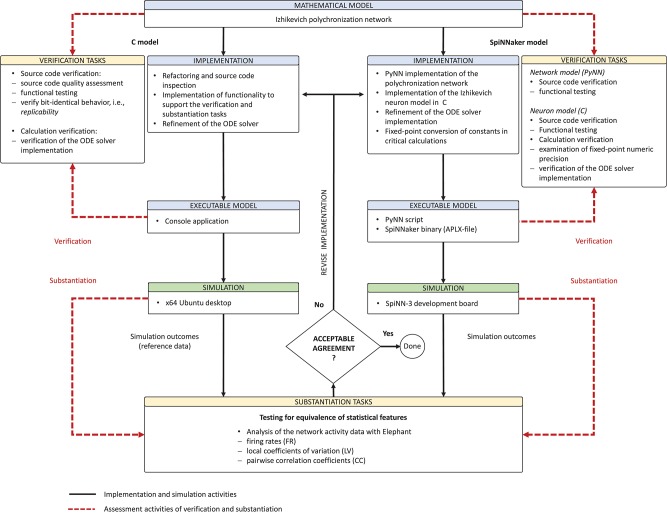
Model verification and substantiation workflow as it was conducted. The figure depicts in a condensed form the instantiation of the model verification and substantiation workflow (Figure 2) introduced in section 2.3 and carried out in this study.

Derived from the concept of model verification and substantiation (Figure 2), the workflow in Figure 5 depicts a condensed illustration of the activities performed in this study. We execute the workflow several times whilst subjecting the C and SpiNNaker model implementations to various implementation and verification activities. The latter can be divided into two categories: source code verification and calculation verification.

The purpose of source code verification is to confirm that the functionality it implements works as intended (Thacker et al., 2004). Unlike commercially developed production software, scientific source code is used to draw scientific conclusions and, thus, it should act as an available reference (Benureau and Rougier, 2017).

The purpose of calculation verification is to assess the level of error that arise from various sources of error in numerical simulations as well as to identify and remove them. The types of errors that can be identified and removed by calculation verification are, e.g., errors caused by inadequate discretization and insufficient grid refinement as well as errors by finite precision arithmetic. Insufficient grid refinement is typically the largest contributor to error in calculation verification assessment (Thacker et al., 2004).

### 3.4. Application of the method

**Figure 6:**
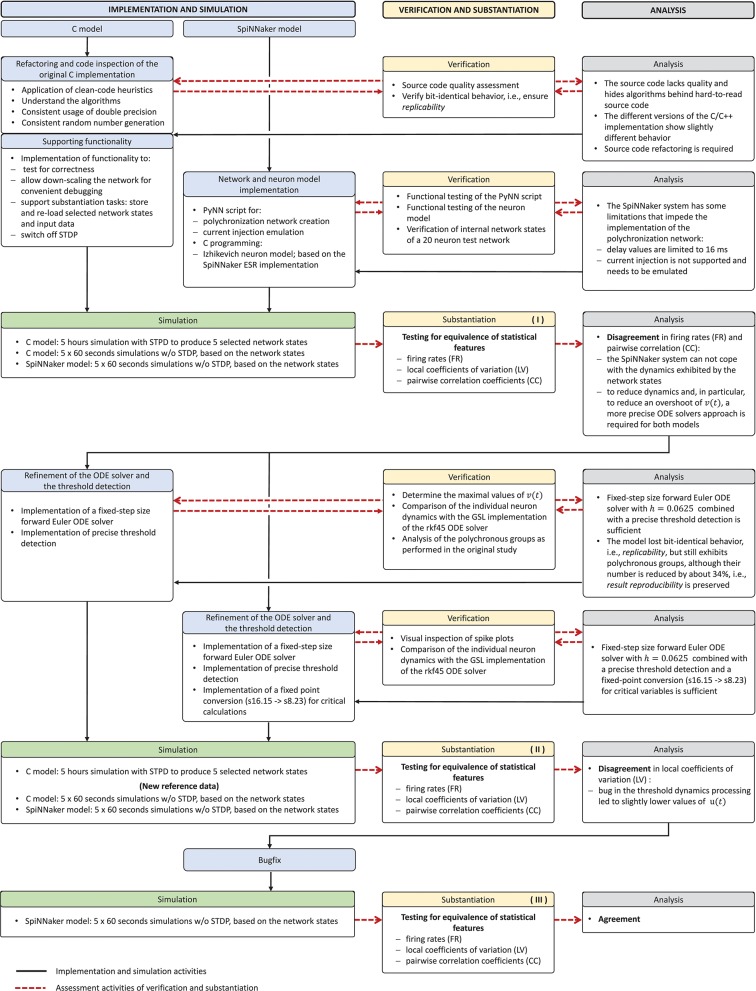
Model verification and substantiation iterations and activities conducted. The activities carried out as part of the model verification and substantiation process, which we briefly outlined in Figure 5, can be further broken down to a more detailed view. The diagram represents this iterative process in a linear fashion, where three iterations have been conducted. The model substantiation activity performed at the end of each iteration is marked with I, II, and III, which corresponds to the results summary shown in Figure 7.

The model verification and substantiation process we describe in this study required three iteration cycles, named Iteration I, II, and III, until an acceptable agreement was achieved. Figure 6 shows a complete and detailed breakdown of the activities, which were shown in more general form in Figure 5.

**Figure 7:**
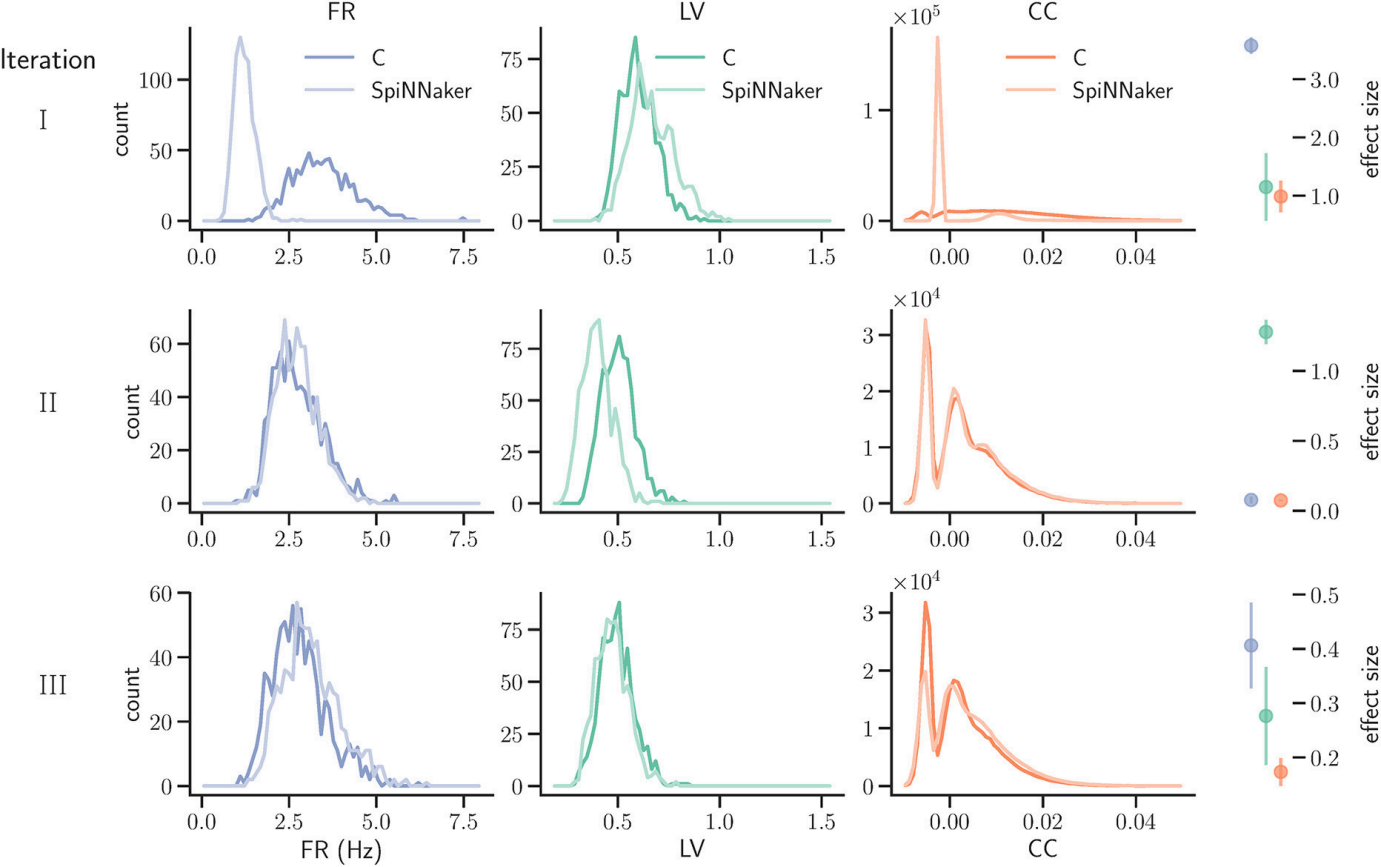
Model substantiation assessment based on spike data analysis. Histograms (70 bins each) of the three characteristic measures computed from 60 s of network activity after the fifth hour of simulation: Left, firing rates (FR); middle, local coefficients of variation (LV); right, pairwise correlation coefficients (CC). For FR and LV, each neuron enters the histogram, for CC each neuron pair. Results are shown for three iterations (rows) of the substantiation process of the C model (dark colors) and SpiNNaker model (light colors), cf. Figure 6. On the far right, the difference between the respective distributions is quantified by the effect size: the graph shows the mean and standard deviation effect size calculated for each of the five network states (after 1, 2, 3, 4, and 5 h of simulation).

In the following, we describe for each iteration the verification activities that identified issues with the executable models, and the consequent adaptations to the C and SpiNNaker model implementations. The substantiation activity performed at the end of each iteration is marked in Figure 6 with I, II, and III, respectively; the results for each one are given in Figure 7. A full description of these and further substantiation activities is provided in our companion study (Gutzen et al., 2018).

In order to be able to reproduce the findings of this work and our companion study (Gutzen et al., 2018), all source code and simulation data is available online. The model source codes, simulation scripts and the codes used in the verification activities are available on GitHub^8^ (doi: 10.5281/zenodo.1435831). The simulation data and scripts used for the quantitative comparisons of statistical measures in the substantiation task can be found on GIN^9^.

#### 3.4.1. Iteration I

In the first iteration, our main focus is source code verification. For the C model, this takes the form of assessing and improving source code quality, whereas for the SpiNNaker model implementation we carry out functional testing.

##### 3.4.1.1. C model

The *poly_spnet.cpp* source code hides the algorithms—which seem to be derived from MATLAB programming paradigms—behind hard-to-read source code. To improve the readability, understand the algorithms, and find potential programming and implementation errors, we subjected the source code to a refactoring^10^ and code inspection task.

We fully reworked the source code by following clean code heuristics (Martin and Coplien, 2009). Code sections concerned with the analysis and not part of the model itself were removed from the source code, kept separately and were only used for functional testing. Whilst going through this iterative refactoring and code inspection process, we made sure that the model remained bit-identical after every iteration, i.e., ensuring replicability (see section 2).

In order to support the experimental setup and make the substantiation activities possible, we added functionality that allows network states to be saved and reloaded. For producing the network activity data for use in substantiation, i.e., the quantitative comparisons of statistical measures, we also switched off STDP (see also section 3.2.1). For convenient functional testing and debugging purposes, the implementation was adapted to allow the polychronization model to be down-scaled to a 20 neuron test network. This size was selected to be small enough for convenient manual debugging, whilst large enough to exhibit spiking behavior and have a non-trivial connectivity matrix.

Performing the refactoring task not only helped understand the C model implementation and algorithms, which is essential, it also laid the foundation for the implementation of the SpiNNaker model.

##### 3.4.1.2. SpiNNaker model

For the initial iteration of the SpiNNaker model, we used the Explicit Solver Reduction (ESR) implementation of the Izhikevich model provided by the SpiNNaker software stack (Hopkins and Furber, 2015). For network creation, simulation control and execution as well as for functional testing, we developed PyNN scripts that allowed us to conveniently perform the simulation, the verification tasks, and substantiation activities. Additional development work was required to circumvent a few restrictions of the SpiNNaker system and its software stack, namely:

***The SpiNNaker framework does not allow external current injection:***During each 1ms simulation time-step, an external current of *I*_ext_ = 20pA is injected into a randomly selected neuron. This current injection is emulated by two spike source arrays forming one-to-one connections to the two populations of the polychronization network. Those connections use static synapses, translating an external spike event into an injected current.

***The amount of data that needs to be held on the*** ***SpiNN-3*** ***board during simulation may*** ***become too large for 60 s simulation time:***To limit the amount of data, we divided a single simulation run into 60 cycles. At the end of each cycle, the simulation is halted for data exchange, and then resumed.

We used three approaches to functionally test the PyNN scripts and to verify the implementation of the neuron model:

***Manual low level debugging on the SpiNNaker system to verify the correctness of state*** ***variables, program flow and algorithms:***The SpiNNaker system offers a low level command line debugging tool called *ybug* and the SpiNNaker kernel also allows log information to be sent to an internal i/o-buffer. The buffer is read at simulation termination and accessible with *ybug*. We used this basic debugging technique to verify the internal states of the neuron model, the correctness of injected current values as well as to verify the correctness of the program flow of the algorithms that we implemented.

***Verification of the neuron dynamics using a PyNN test script applying an external*** ***constant current to individual neurons and recording the state variables:***We recorded the dynamics of individual neurons resulting from an injected constant current and compared the data with the results obtained from a stand-alone C console application that implements the same algorithms.

***Functional testing with a small (20 neuron) version of the polychronization network:***We used a down-scaled version of the polychronization network (16 excitatory and 4 inhibitory neurons) to verify the functional correctness of the simulation setup. As the connectivity matrix was derived from simulations of the C model, it further served for testing the functionality added to support the activities carried out during the substantiation process, e.g., the export of the connectivity matrix created by simulation runs of the C model and its import into the SpiNNaker simulation.

##### 3.4.1.3. Substantiation

We simulated the models to generate the data for the quantitative comparisons of the statistical measures, as described in sections 3.2.1 and 3.2.2, respectively. The results are summarized in the top row of Figure 7. This reveals a substantial mismatch, most dominantly visible in the distribution of the firing rates (FR) and the pairwise correlation coefficients (CC). This mismatch, as quantified by the effect size, is consistently observed for all five reference network states. Therefore, we conclude that the models do not show an acceptable agreement and the substantiation assessment failed at the end of Iteration I. Although the effect size is a very simple measure which only takes into account the means and standard deviations of the distributions, it provides an intuitive quantification of differences which is unbiased by the sample size. However, since the effect size can not detect discrepancies in the distribution shape, a visual inspection is essential and additional comparison methods, such as hypothesis tests, may be needed. In Figure 7 we only show the measures computed from 60 s of network activity after the fifth hour. For a visual inspection of the computed measures from the network states after 1, 2, 3, 4, and 5 h of simulation, see Figures S1–S5 in the Supplementary Material.

#### 3.4.2. Iteration II

The substantial discrepancies revealed by the model substantiation assessment performed in Iteration I suggests that there are numerical errors in one or both of the executable models. In the second iteration, we therefore focus on calculation verification. To this end, monitoring functionality was included to record the minimal, maximal, and average values of the model state variables. We find that the largest contributors to error are the choice of solver for the neuronal dynamics, the detection of spikes, and the fixed-point arithmetic on SpiNNaker.

##### 3.4.2.1. Numeric integration scheme and precise threshold detection

When working with systems of ordinary differential equations (ODEs), it is important to make sensible decisions regarding the choice of a numeric integration scheme. To achieve accurate approximations of their solutions one must take into account not only the form of the equation but also the magnitude of the variables occurring in them (Dahmen and Reusken, 2005). Depending on these parameters, some ordinary differential equations can become *stiff* , i.e., requiring excessively small time steps for an *explicit* numerical iteration scheme (i.e., one that only uses the values of variables at preceding time-steps) to achieve acceptable accuracy and avoid numeric instabilities. Such equation systems require the use of an *implicit* scheme (i.e., one that finds a solution by solving an equation involving both the current values of variables and their later values). However, this method is computationally more expensive, entailing unnecessarily long run-times when applied to non-stiff systems (Strehmel and Weiner, 1995). The ODEs used to model neuronal behavior are often non-stiff, so that an explicit numerical iteration scheme is sufficient (Lambert, 1992).

The Izhikevich ODE system (Equations 1–3) is an example of such a non-stiff model, see Blundell et al. (2018b). Thus, in principle, the choice of an explicit method, namely the Forward Euler method, albeit in a semi-implicit symplectic variant, which is used in the C model, is correct. Nevertheless, the numerical integration scheme must be applied correctly, i.e., the step size must be chosen according to the desired maximum error. The (relatively large) selected step sizes of *h* = 0.5ms for the integration of the membrane potential (Equation 1), and *h* = 1.0ms for the recovery variable are not only questionable because no motivation is given for why two different step sizes are chosen for the same system of equations, but more importantly because no error estimate is implemented to guarantee that the integration scheme does in fact give a reasonable approximation of the solution of the ODE system. The algorithm of the original C model implementation is shown in Listing 1. Note the symplectic, or semi-implicit Forward Euler scheme, i.e., the update of *u* is based on an already updated value for *v*. In an unorthodox approach, the variable v is integrated in two 0.5ms steps whilst u is integrated in one 1ms step.

**Listing 1:**
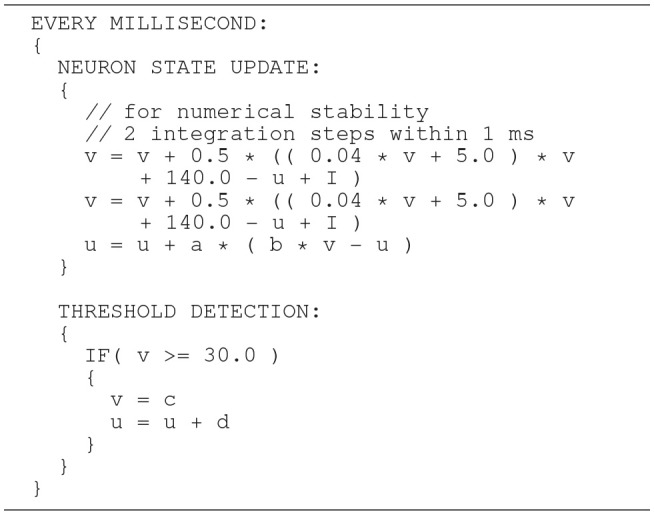
C model: algorithm of updating the neuronal dynamics (given as pseudocode) as implemented in the original C model. The algorithm implements a fixed-step size semi-implicit symplectic Forward Euler method.

**Figure 8:**
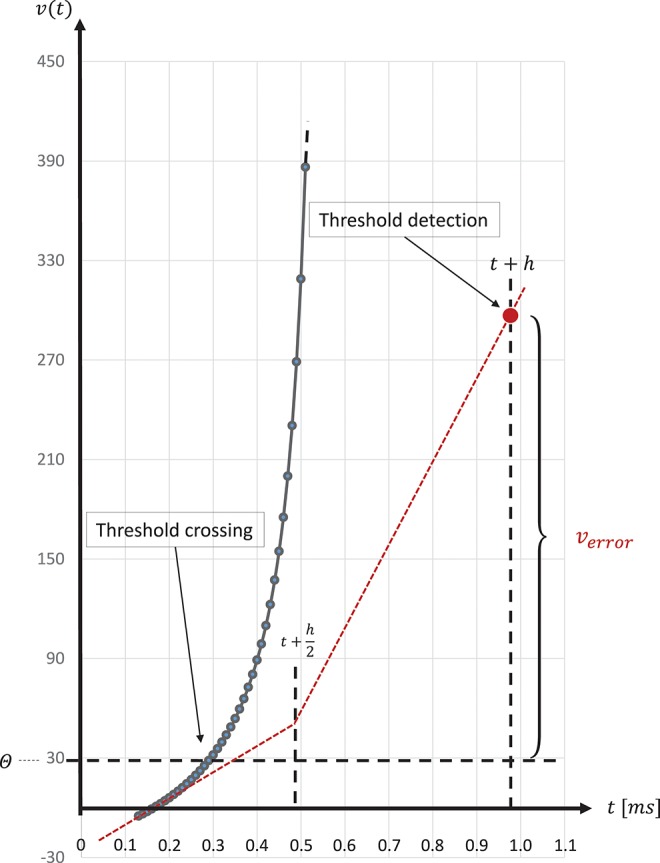
Above threshold evolution of the state variable *v*(*t*). The approximation in the evolution of *v*(*t*) in the Equation (1) when using the semi-implicit symplectic Forward Euler method with a fixed-step size of *h*/2 = 0.5ms (the red dotted line), where *h* refers to the 1 ms simulation time-step, causes *v*(*t*) values to be well above the threshold and, thus, producing a propagating error over time. This is expressed in delayed spike times. The black solid line shows the evolution of *v*(*t*) around threshold for a regular-spiking type Izhikevich neuron stimulated with a constant current of *I*_ext_ = 5pA. For integration, the same Forward Euler method was used but with an integration step size of *h*/100 = 0.01ms. The steep slope at threshold requires a precise threshold detection to prevent a numeric overflow.

The spike onset of a regular-spiking Izhikevich neuron appears as a steep slope at threshold, and, due to the grid-constrained threshold detection in the C model, leads to values of *v*(*t*) which can be two orders of magnitude higher than the threshold value θ = 30mV (Equation 3). In the C model, we observed values of *v*(*t*) ≤ 1700. Figure 8 graphically illustrates the error caused by this approximation. The value of *u*(*t*) (Equation 2), which describes the threshold dynamics, evolves continuously, thus, *v*_error_ will induce an error to the threshold dynamic which propagates over time delaying all subsequent spike events.

**Figure 9:**
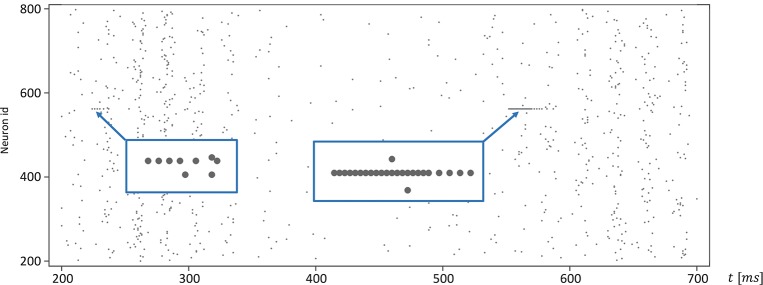
Spike artifacts caused by fixed-point overflow. Large values of *v*(*t*) can cause an overflow of the fixed-point data type, which may result in short spike-trains with higher rates (marked by blue boxes). Simulations on SpiNNaker using fixed-step size symplectic Forward Euler with an integration step size of *h*/3 = 0.333ms and without precise threshold detection. (*h* refers to the simulation time-step of 1ms).

Moreover, for efficiency, SpiNNaker uses fixed-point numerics. Numbers are held as 32-bit fixed-point values in a *s*16.15 representation, limited in range. Large values of *v*(*t*) can lead to a fixed-point overflow, as discussed in greater detail below, which may then produce spike artifacts. The likelihood of this is even further increased by the fact that this value appears as a power of two in Equation (1). To demonstrate this, we adapted the algorithm shown in Listing 1 and added an additional integration step (see Listing 2). The neuronal activity, shown in Figure 9, exhibits spiking artifacts in the form of bursts of spikes with high spike rates.

**Listing 2:**
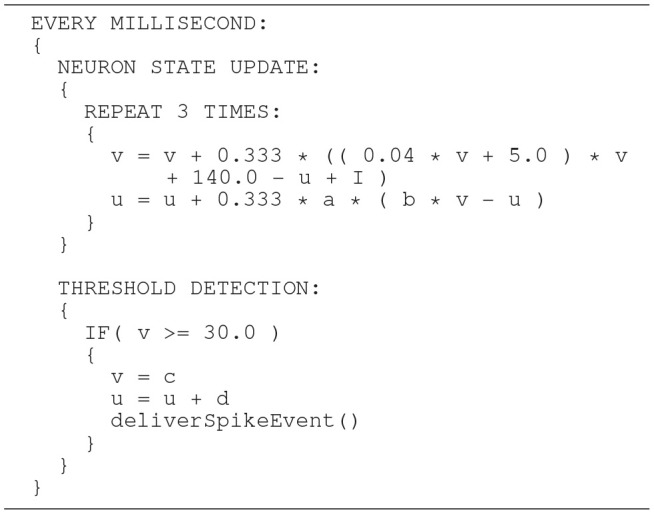
SpiNNaker model: an algorithm of updating the neuronal dynamics (given as pseudo code). The algorithm is similar to the implementation shown in Listing 1 but uses three fixed size integration steps. The additional step increases the likelihood that large values of *v*(*t*) are squared. This implementation may cause a numeric overflow.

The SpiNNaker software stack (Rowley et al., 2017b) provides an Izhikevich neuron model implementation optimized for efficiency for fixed-point processors, such as ARM. The implementation follows a new approach called Explicit Solver Reduction (ESR), described in Hopkins and Furber (2015): “*for merging an explicit ODE solver and autonomous ODE into one algebraic formula, with benefits for both accuracy and speed.”* The SpiNNaker system is designed for simulations in biological real-time. The real-time capability is achieved at an integration step size of *h* = 1ms which then corresponds to the simulation time-step, i.e., the same integration step size as the C model. At higher resolution, i.e., smaller integration time-steps, the simulation time increases accordingly. The SpiNNaker ESR implementation, at the same integration step size, does not exhibit such artifacts, but fails in in adequately reproducing the network states, as can be seen in the model substantiation assessment for Iteration I (top row of Figure 7).

In general, higher accuracy can be obtained by using smaller step sizes. However, for this model, using smaller steps to integrate whilst restricting spike detection and reset to a 1ms grid results in a steep slope in the evolution of the membrane potential above threshold which rapidly reaches values that can not be represented with double precision (compare red dotted curve and black solid curve in Figure 8). We therefore propose a solution that combines a simple fixed-step size symplectic Forward Euler ODE solver and an exact off-grid threshold detection, while a spike event is still forced to a grid point. To be more specific, within each 1 ms simulation time-step *h*, the equations evolve in steps of *h*/16. The number of internal integration steps was chosen for two reasons. First, as a power of two, it can be represented in *s*16.15 without numerical error. Second, it represents a good compromise between the increased computational cost of smaller steps, and the increased overshoot in the membrane potential for larger steps. The algorithm is given as pseudo code in Listing 3. Please note the multiplication with 0.0625, avoiding a costly division. Spikes can be detected (and the dynamics reset) after every internal step, however, as with the C model, spikes are emitted on the simulation grid with a resolution of 1ms. Multiple spike events within one simulation time-step are thus potentially possible, but are merged into a single event. However, this seems to be a very rare event. Pauli et al. (2018) demonstrated that there was only a very slight change in average firing rate for this network model between a simulation locked to a 1ms grid, as used here, and one carried out at a higher resolution of 0.1ms. We thus consider this effect to be negligible in the following.

**Listing 3:**
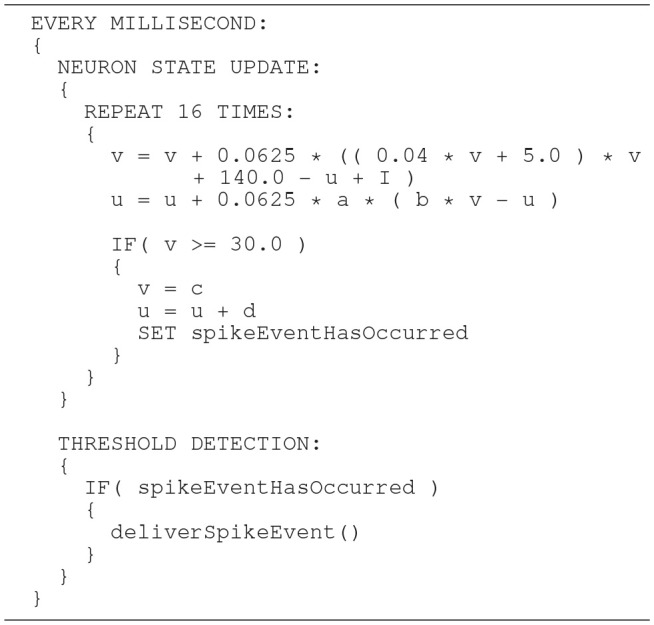
SpiNNaker model: an improved algorithm of updating the neuronal dynamics (given as pseudo code) that uses a fixed-step size symplectic Forward Euler method and precise threshold detection.

**Figure 10:**
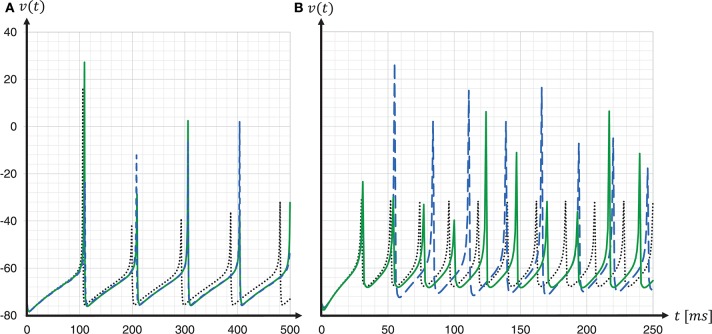
Spike timing: comparison of different ODE solver implementations. Membrane potential *v*(*t*) recorded for a regular-spiking **(A)** and fast-spiking **(B)** Izhikevich neuron, stimulated with a constant current of *I*_ext_ = 5pA. The dynamics are solved by the original SpiNNaker ESR ODE solver implementation (blue dashed curves); a fixed-step size symplectic Forward Euler approach with precise threshold detection (*h*/16 = 0.0625ms) (green solid curves); and, for comparison, a reference implementation of the GSL rkf45 ODE solver with an absolute integration error of 10^−6^ (black dotted curves). Both the SpiNNaker ESR and the fixed-step size Forward Euler implementations show considerable lags in the spike timing compared to the rkf45 reference implementation. While for the regular-spiking neuron **(A)** the SpiNNaker implementations have much the same accuracy, the fixed-step size Forward Euler approach with precise spike timing shows a substantial improvement over the ESR implementation for the fast-spiking neuron **(B)**.

To assess the accuracy of our proposed solver and that of the implementation provided by the SpiNNaker framework, we performed single neuron simulations and compared the resultant membrane potentials to that produced by a Runge-Kutta-Fehlberg(4, 5) (rkf45) solver implementation from the GNU Scientific Library (GSL)^11^. The explicit Runge-Kutta-Fehlberg(4, 5) method is a good general-purpose integrator, and, compared to a simple Forward Euler, of a higher order. To serve as a reliable reference, the rkf45 algorithm was parametrized to integrate with an absolute error of 10^−6^. The results are shown in Figure 10. Note that not only do the spike times for both the fixed-step size Euler and the ESR solvers lag behind the rkf45 solver, but due to the accumulation of *v*_error_, the lag becomes larger during the course of the simulation, here reaching around 20ms in a simulation of 500ms duration containing five spikes. As the errors occur at spike times, higher spike rates lead to larger deviations. Thus, the course of the membrane potential of the fast-spiking type neuron is less accurate than for the regular-spiking type neuron. This applies also to an increasing injected current *I*, as this also leads to higher spike rates (data not shown). As the firing rate increases, the ESR lags more, such that fewer spikes are generated in the given time window. Our results show that even though the fixed-step size Euler scheme is simpler than ESR, it is a more accurate match to the single neuron dynamics.

##### 3.4.2.2. Fixed-point numeric precision

Hardware floating point units are expensive in chip area, and thus lower the power efficiency of the system. Consequently, SpiNNaker stores numbers, i.e., membrane voltages and other neuron parameters, as 32-bit signed fixed-point values (Furber et al., 2013). Since the meaning of an *n*-bit binary word depends entirely on its interpretation, we can divide an n-bit word into an integer part *i* and a fractional part *f* by defining a *binary point* position. Calculations are then performed as if the numbers are simple two's complement integers. SpiNNaker uses a so called *s*16.15 representation, that is, a 32-bit signed fixed-point format with *i* = 16, *f* = 15 and a sign bit. The value range is small in comparison to a single or double precision data type. For the *si*.*f* data types the value range is defined by:

(5) $$\begin{array}{r} -2^{i}\leq x\leq +2^{i}-2^{-f}. \end{array}$$

The SpiNNaker *s*16.15 data type therefore ranges from −2^16^ = −65536 to 2^16^−2^−15^ = 65535.999969482.

This data type does not saturate on SpiNNaker (Hopkins and Furber, 2015). This means that in case of a fixed-point overflow, the value wraps around producing a negative number. In neural network simulations this might be seen as spike artifacts, as demonstrated in Figure 9. Another aspect of fixed-point arithmetic and an additional source of numerical inaccuracy is that not every number can be accurately represented. For example: although small, the error in the *s*16.15 representation of the constant value 0.04 in Equation (1) induces a noticeable delay in the spike timing.

To represent a number in *si*.*f*, its value is shifted *f* bits to the left, i.e., multiplied by 2^*f*^. For the constant value 0.04 in Equation (1) this yields:

$$\begin{array}{l} 0.04\cdot 2^{15}=1310.72_{\left(s16.15\right)} \end{array}$$

The compiler stores the value as a 32-bit word while truncating the fraction:

$$\begin{array}{l} 0x0000051E \end{array}$$

If the value is converted back, this leads to:

$$\begin{array}{l} 1310_{\left(s16.15\right)}\cdot 2^{-15}=0.03997802 \end{array}$$

This loss in precision is significant. At the level of the dynamics of individual neurons, this difference is expressed in terms of delayed spike times. The following example may illustrate this: for the sake of simplicity we assume a membrane potential of *v*(*t*_0_) = −75mV while *u*(*t*_0_) = 0 and *I*(*t*_0_) = 0. The expected value for *v*(*t*_1_) in the Equation (1) is:

$$\begin{array}{l} 0.04\cdot 75\cdot 75+5\cdot \left(-75\right)+140=-10.0000000 \end{array}$$

The same calculation in *s*16.15 leads to:

$$\begin{array}{l} 0.03997802\cdot 75\cdot 75+5\cdot \left(-75\right)+140=-10.1236357 \end{array}$$

This slightly more negative value of *v*(*t*) causes the threshold crossing to occur later and affects the dynamics on the network level.

The effect can be mitigated if critical calculations are performed with higher precision numbers, whereby the order of operations also plays a role. If, for example, the constant value 0.04 in Equation (1) is represented in *s*8.23, the numerical error can be reduced.

$$\begin{array}{l} 0.04\cdot 2^{23}=335544.32_{\left(s8.23\right)} \end{array}$$

If the value which is truncated by the compiler is converted back, we then get:

$$\begin{array}{l} 335544_{\left(s8.23\right)}\cdot 2^{-23}=0.039999962 \end{array}$$

If now the same calculation as in the beginning is performed, the result is significantly more precise.

$$\begin{array}{l} 0.039999962\cdot 75\cdot 75+5\cdot \left(-75\right)+140=-10.00021375 \end{array}$$

The disadvantage, however, is the limited value range of the *s*8.23 representation which is:

$$\begin{array}{l} -2^{8}=-256\text{ to }2^{8}-2^{-23}=255.999999881 \end{array}$$

The simple fixed-step size symplectic Forward Euler method together with a precise threshold detection presented above ensures that values stay within limits. Furthermore, we point out that a *s*8.23 data type is not available on SpiNNaker, i.e., it is not supported by the ARM C compiler. To let the value 335544.32_(*s*8.23)_ appear as a *s*16.15 constant we can write:

$$\begin{array}{l} 335544.32_{\left(s16.15\right)}=10.24\cdot 2^{15} \end{array}$$

In order to return to the original value, a right-shift operation of 8 bits is then required.

$$\begin{array}{l} 10.24\cdot 2^{-8}=0.04 \end{array}$$

In this context, the order in which the operations are carried out is also very important. For example, multiplying 10.24 with the power of two of the membrane potential may cause an overflow of the *s*16.15 data type. Combining all this leads to the following sequence of operations for the Equation (1).

(6) $$\dot{v}=((10.24\cdot v)\cdot 0.00390625))\cdot v+5\cdot v+140-u+I$$

In order to prevent the compiler from optimizing the code and perhaps arranging the operations in an inappropriate order, the critical calculations in the Equation (6) are placed in separate lines. This is shown as pseudo code in Listing 4. Note that suppressing optimization in this way works for the ARM C compiler, but can not be generalized. We verified this through an analysis of the generated assembler source code.

**Listing 4:**
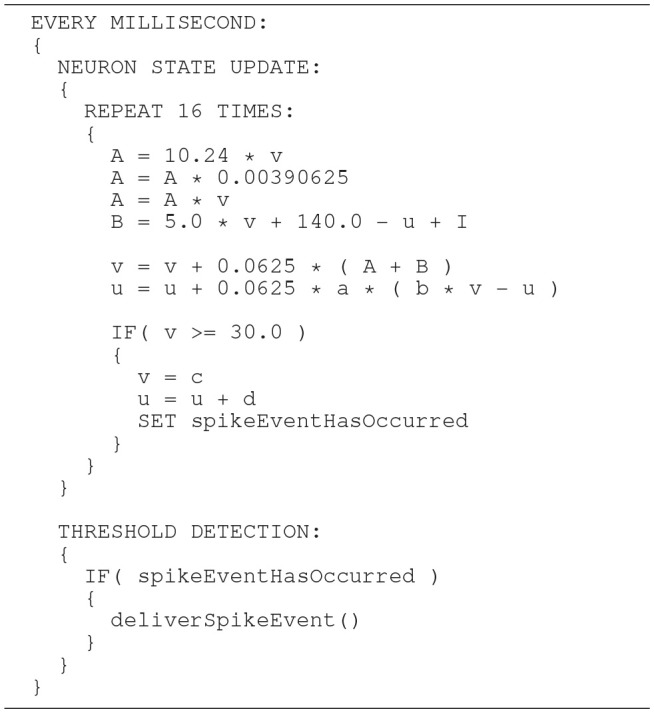
SpiNNaker model: the same algorithm (given as pseudo code) as shown in Listing 3, but adds fixed-point conversion to the constant 0.04.

The above also applies to the Izhikevich neuron model parameters *a* and *b* which add an error to *u*(*t*). Further, the example ignored that the state variables *v*(*t*) and *u*(*t*) are themselves fixed-point values that add numerical inaccuracy.

In the course of the implementation of the SpiNNaker Izhikevich neuron model, and the adaptations of the model during the verification and substantiation process, we added fixed-point data type conversion to all constant values involved in critical calculations, that is the constant value 0.04 in the Equation (1) and the neuron model parameters *a* and *b*.

**Figure 11:**
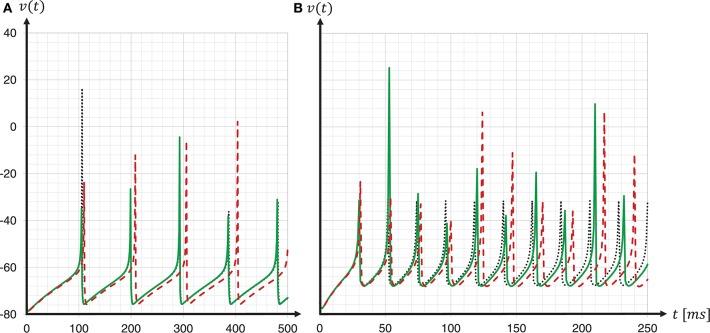
Spike timing: with and without fixed-point data type conversion. The graphs show the development of the membrane voltages *v*(*t*) with (green solid line) and without (red dashed line) fixed-point data type conversion for a regular-spiking type **(A)** and a fast-spiking type **(B)** Izhikevich neuron, that is stimulated with a constant current of *I*_*ext*_ = 5*pA*. For the ODE solver, the fixed-step size symplectic Forward Euler implementation with precise threshold detection was used (*h*/16 = 0.0625ms). This is shown in comparison to a reference implementation of the GSL rkf45 ODE solver with an absolute integration error of 10^−6^ (black dotted line). For both neuron types, a substantial improvement in the spike timing can be seen.

To investigate the consequences of data type conversion for critical parameters on the accuracy of the solution of the dynamics, we simulated regular-spiking and fast-spiking Izhikevich neurons with and without fixed-point data type conversion, and compared the development of the membrane voltages to a Runge-Kutta-Fehlberg(4, 5) (rkf45) solver implementation of the GNU Scientific Library (GSL), thus, using the same verification method as before when choosing the integration scheme. The results are shown in Figure 11. For both neuron parameterizations, we achieved a substantial improvement in the spike timing. Compared to results for the regular-spiking neuron, in which the solver employing data type conversion is very close to the rkf45-reference, our implementation still lags behind the rkf45-reference for the fast-spiking neuron. This can be explained by the overshoot in *v*(*t*) at threshold crossing, that, even if it is small, still exists, and propagates over time—and the more spikes emitted, the larger the error becomes.

##### 3.4.2.3. Substantiation

As the C model was adapted during Iteration II, we can no longer speak of a *replication*. Therefore, before performing the model substantiation assessment, we needed to check whether the results of the modified model are compatible with the original, i.e., whether or not *result reproducibility* is preserved. We evaluated the development of polychronous groups in the modified C model using the analysis provided in Izhikevich (2006). We found that the number of polychronous groups was reduced by about 34%. Thus the network still shows the behavior reported in the original manuscript (Izhikevich, 2006), albeit in a weakened form. As it was demonstrated in Pauli et al. (2018) that the number of groups developed by the C model varies strongly with implementation details, including the solver algorithm of the neuron model, we consider this result to be within our acceptance criteria.

We then performed the model substantiation assessment as described in section 3.2 for the C and SpiNNaker models incorporating the refined neuron model implementations described above. Note that this included re-generating the reference data, due to the changes in the neuron model implementation.

The result of the network activity data analysis and its comparison is shown in the middle row of Figure 7. Our new ODE solver, implemented in both models, leads to a good match in the firing rates (FR) and the pairwise correlation coefficients (CC). We note, though, that the distributions are shifted from those expressed by the C implementation in Iteration I. The shift of cross-correlation to lower values may well account for the smaller number of polychronous groups developed. Both the firing rates and the cross correlations also show small effect sizes after this iteration. In case of the CC distributions, the effect size has to be interpreted with care, as it assumes Gaussian-like distributions which is clearly violated by the bimodality of the CC distributions. Nevertheless, in combination with visual inspection and additional comparison measures, its application here provides a useful discrepancy quantification.

A discrepancy can still be seen between the distributions of the coefficients of variation (LV). The distribution for the SpiNNaker model is shifted toward lower values, indicating a higher degree of regularity than that of the C model. This is confirmed by the consistently high effect size obtained for the five reference network states. Therefore, we conclude that there is still a disagreement in the executable models, and that model substantiation assessment has not been achieved at the end of Iteration II.

#### 3.4.3. Iteration III

The slight discrepancy in regularity observed in Iteration II allowed us to identify systematic differences in spike timing between the two models, hinting at an error in the numerical integration of the single neuron dynamics. Indeed, the visual comparison of the dynamics of individual neurons on SpiNNaker with a stand-alone C application that implements an identical fixed-step size symplectic Forward Euler ODE solver, revealed a small discrepancy in the sub-threshold dynamics, leading to a fixed delay in the spike timing. We identified an issue in the precise threshold detection algorithm as to be the cause.

##### 3.4.3.1. Substantiation

The result that we achieved after resolving the issue and repeating the SpiNNaker simulations is shown in the bottom row of Figure 7. We observe a close match of all three distributions, consistently across the five reference network states. The comparison is not perfect, with the distribution of firing rates showing the largest discrepancy with only a subtle shift toward higher firing rates for the SpiNNaker simulation. The small discrepancies between the two implementations are quantified by the effect size, and demonstrate that we have achieved a considerable reduction of the mismatch as a result of the model verification and substantiation process. All effect sizes are classified in the range of small to medium according to Cohen (1988). While further iterations of the model implementation in the verification and substantiation process (see section 4 for suggestions) may further improve the effect size scores, for our purposes, we find the remaining mismatch in the range of acceptable agreement. We therefore conclude that the executable models are in close agreement at the end of Iteration III.

## 4. Discussion

In this study, we introduced the concept of model verification and substantiation. In conjunction with the work presented in Gutzen et al. (2018), we demonstrated the application of a rigorous workflow assessing the level of agreement between the C implementation of the spiking network model proposed by Izhikevich (2006) and a reproduction of its underlying mathematical model on the SpiNNaker neuromorphic system. The choice of this network was motivated by its unorthodox implementation choices, examined in greater detail in Pauli et al. (2018). These issues make it a particularly illustrative example for a reproduction on the SpiNNaker neuromorphic system and to demonstrate various aspects of source code and calculation verification.

After three iterations of the proposed workflow we concluded, on the basis of the substantiation assessment, that the executable models are in acceptable agreement. This conclusion is predicated on the domain of application and the expected level of agreement that we defined for three characteristic measures of the network activity. We emphasize that these definitions are set by the researcher: further iterations would be necessary, if, for example, we set a level of agreement requiring a spike-by-spike reproduction of the network activity data, as applied by Pauli et al. (2018).

We speculate that the remaining mismatch in the statistical measures at the end of Iteration III can be explained by the reduced precision in the representation of the synaptic weights on the SpiNNaker system. This source of error is introduced by the conversion of the double precision weight matrix exported from the C model and converted into a fixed-point representation when imported into the simulation on the SpiNNaker system. The absolute values of the synaptic weights after conversion are always smaller than its double origin, thus, negative weights increase, contributing to larger firing rates on SpiNNaker (see Iteration III in Figure 7). Another potential source of error, in terms of calculation verification, is related to the grid based simulation paradigm, i.e., the simulation time-step, with which spike events are delivered. Both the original C model implementation and the SpiNNaker system use a simulation time-step of 1ms, which is larger than commonly used in spiking neural network simulations. Since both models are affected, the substantiation assessment can not give us further insight.

Although some of the verification tasks we applied, such as functional testing, are closely tied to model implementation details, the methodology presented in this work is transferable to similar modeling tasks, and could be further automated. The quantitative comparison of the statistical measures carried out in the substantiation was performed using the modular framework NetworkUnit^12^ (NetworkUnit, RRID:SCR_016543), an open source Python module, presented in the companion study to this work (Gutzen et al., 2018). NetworkUnit facilitates the formalized application of standardized statistical test metrics that enable the quantitative validation of network models on the level of the population dynamics.

The model substantiation methodology we propose has a number of advantages. Firstly, from the point of view of computational neuroscience, simulation results should be independent of the hardware, at least on the level of statistical equivalence. In practice, implementations may be sensitive to issues such as 32/64-bit architecture or compiler versions. Thus, the underlying hardware used to simulate a model should be considered part of the model implementation. Applying our proposed model substantiation methodology allows a researcher an opportunity to discover and correct such weaknesses in the implementation. Secondly, in the case of new types of hardware, such as neuromorphic systems, the methodology used here can help to build confidence and uncover shortcomings. In the particular example investigated here, we were able to demonstrate that the numerical precision is a critical issue for the model's accuracy. Integrating the model dynamics at 1ms resolution using 32-bit fixed-point arithmetic available on SpiNNaker (Furber et al., 2013) does not adequately reproduce the dynamics of the corresponding C model with floating point arithmetic. We propose an alternative integration strategy that does adequately reproduce the dynamics, but the more general point is that this study demonstrates how the use of a rigorous model substantiation methodology can contribute to fundamental open questions in neuromorphic computing, such as the required level of precision in the representation of variables. Finally, in neuroscience, models often function as discovery tools and hypothesis generators in cases where experimental data, against which a model could be validated, does not exist. Performing a substantiation assessment is an option to accumulate circumstantial evidence for a model's plausibility and self-consistency, although it cannot reveal whether a model reflects reality.

Beyond our introduction of the term *substantiation*, we have adopted the ACM (Association for Computing Machinery, 2016) terminology for reproducibility and replicability, as it seems most appropriate for our purposes. Alternative definitions exist, and terminology for research reproducibility is an ongoing theme of a controversial debate. The application of methodologies from model verification and validation (Thacker et al., 2004) to the field of neural network modeling and simulation can be of great value, but we have suggested some adaptations that, in our view, fit the domain better. In particular, the terms *mathematical model* and *executable model*, that we propose instead of using the terms *conceptual model* and *computerized model*, are intended to yield better separation of the entities they describe, so that, for example, implementation details are not falsely understood to belong to the mathematical model. This is important, as the classic “one model—one code” relationship does not typically apply to spiking neuron network models. Instead, they are implemented using general purpose neural simulation tools such as NEURON (Hines and Carnevale, 1997), Brian (Goodman and Brette, 2008), or NEST (Gewaltig and Diesmann, 2007), which can run many different models. In addition, model simulation codes may be partially generated by other tools (Blundell et al., 2018a). This scenario abstracts the implementation details away from the modeler, who can focus on analysis and modeling, and has the further advantage that individual components (such as neuron models) can be separately verified, and may subsequently serve as reliable references. We hope that our proposed terminology will help to pave the way to a more formalized approach for model verification and validation in the domain of neural network simulation.

In this study, we applied a number of standard methods from software engineering. This discipline is concerned with the application of a systematic, disciplined, quantifiable approach to the development, operation, and maintenance of software (Bourque and Fairley, 2014). Such methods include, for example, the application of clean code heuristics, test driven development, continuous integration and agile development methodologies, with the common goal of building quality into software. The formalized model verification and substantiation workflow that we presented in this work should be seen in this context.

We note that software engineering methods, whilst critical for developing high quality software, are underutilized in computational science in general, and in computational neuroscience in particular. For the network model investigated here, it is important to emphasize that the awareness of software engineering methodology was even less widespread at the time of publication, and so the yardsticks for source code quality applicable by today's standards should be considered in their temporal distance. Credit must in any case be given for the unusual step of publishing the source code, allowing scientific transparency and making studies such as the current one, and that of Pauli et al. (2018), possible. Following formalized processes, such as the one described here, further aids transparency and comprehensibility, and reduces the risk of incorrect conclusions. Moreover, simulation tools as well as neuromorphic hardware platforms can benefit from formalized and automated verification and validation procedures, such that their reliability can be inherited by user-developed models that are simulated using those tools and frameworks. Most importantly, such standardized procedures are designed not to place an additional burden on researchers, but rather to open up simple avenues for computational neuroscientists to increase the rigor and reproducibility of their models.

In conclusion, we argue that the methods of software engineering, including the model verification and substantiation workflow presented here, as well as verification and validation methodologies in general, need to become a mainstream aspect of computational neuroscience. Simulation and analysis tools, frameworks and collaboration platforms are part of the research infrastructure on which scientists base their work, and thus should meet high software development standards. The consideration of the application of software engineering methodologies to scientific software development should start at the funding level, such that an assessment of the software engineering strategy is part of the evaluation of grant applications. Likewise, journals should become more selective with their acceptance of studies, and reject those for which no demonstration has been made of an attempt to verify the calculations. The use of standard tools goes a significant way to fulfilling this criterion, to the extent that the standard tools themselves are developed with a rigorous testing and verification methodology.

## Author contributions

GT devised the project, the main conceptual ideas and workflows. GT performed the verification tasks, the implementation of the models and their refinement, and performed the numerical simulations. RG and MD designed the statistical analysis which was then carried out by RG. RG and MD have written the passage on analysis of spiking activity and contributed to the terminology section. IB contributed expertise on numeric integration. AM gave scientific and theoretical guidance. GT, RG, MD, and AM established the terminology. All authors provided critical feedback and helped shape the research, analysis, and manuscript.

### Conflict of interest statement

The authors declare that the research was conducted in the absence of any commercial or financial relationships that could be construed as a potential conflict of interest.

## Footnotes

^1^In analog neuromorphic systems the outcome is not only determined by the initial conditions. Chip fabrication tolerances and thermal noise add a stochastic component.

^2^https://www.izhikevich.org/publications/spnet.htm

^3^http://apt.cs.manchester.ac.uk/projects/SpiNNaker/project/Access/

^4^PyNN is a common interface for neural network simulators (Davison et al., 2009).

^5^https://github.com/SpiNNakerManchester/sPyNNaker8NewModelTemplate

^6^This refers to the simulated time and not to the run time of the simulation.

^7^http://neuralensemble.org/elephant

^8^https://github.com/gtrensch/RigorousNeuralNetworkSimulations

^9^https://web.gin.g-node.org/INM-6/network_validation

^10^Refactoring—a software engineering method from the area of software maintenance—is source code transformation which reorganizes a program without changing its behavior. It improves the software structure and the readability, and so avoids the structural deterioration that naturally occurs when software is changed Sommerville, 2015.

^11^https://www.gnu.org/software/gsl/

^12^https://github.com/INM-6/NetworkUnit
