# Supplementary material for: Rigorous Neural Network Simulations: A Model Substantiation Methodology for Increasing the Correctness of Simulation Results in the Absence of Experimental Validation Data
Source: Front Neuroinform. 2018 Nov 26;12:81. doi: 10.3389/fninf.2018.00081 (PMC6275234; doi:10.3389/fninf.2018.00081)
Supplement: Supplementary file 1 [file Data_Sheet_1.PDF]

Supplementary Material: Rigorous neural network simulations: a model substantiation methodology for increasing the correctness of simulation results in the absence of experimental validation data

1 SUPPLEMENTARY FIGURES

The effect size cannot detect discrepancies in the shape of distributions. To demonstrate the stability of the effect size measure in our data, we have collected the computed measures and effect sizes from the network states after 1, 2, 3, 4, and 5 hours of simulation for visual inspection and comparison, corresponding to Fig. S1, S2, S3, S4, and Fig. S5, respectively. The figures show the histograms (70 bins each) of the three characteristic measures computed from 60 s of network activity: Left, firing rates (FR); middle, local coefficients of variation (LV); right, pairwise correlation coefficients (CC). For FR and LV, each neuron enters the histogram, for CC each neuron pair. Results are shown for three iterations (rows) of the substantiation process of the C model (dark colors) and SpiNNaker model (light colors). On the far right, the difference between the respective distributions is quantified by the effect size.

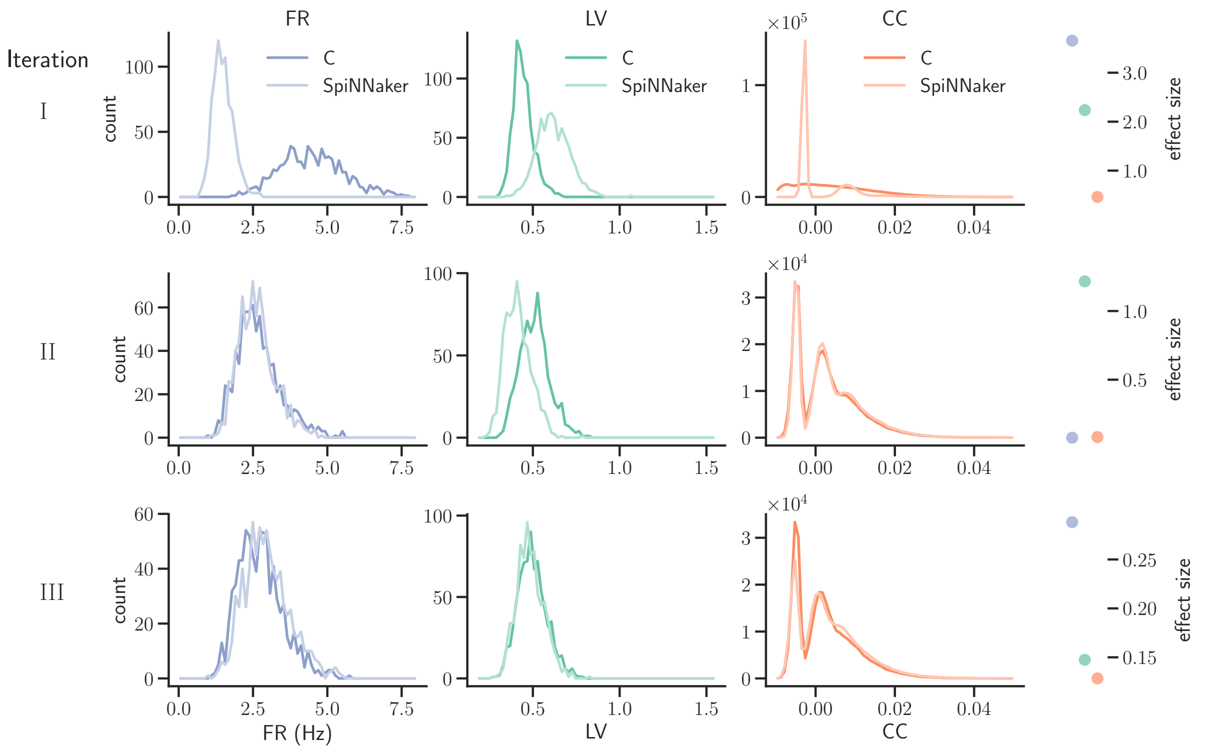

Figure S1. Characteristic measures computed from 60 s of network activity after 1 hour of simulation.

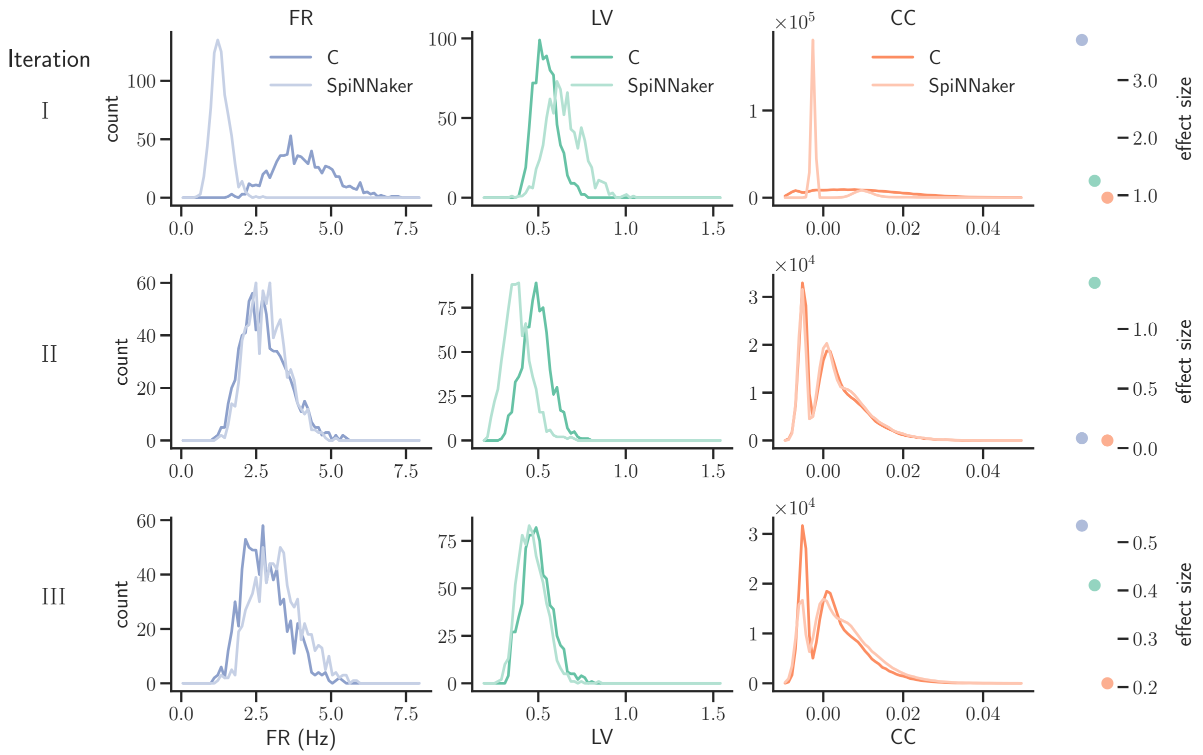

Figure S2. Characteristic measures computed from 60 s of network activity after 2 hours of simulation.

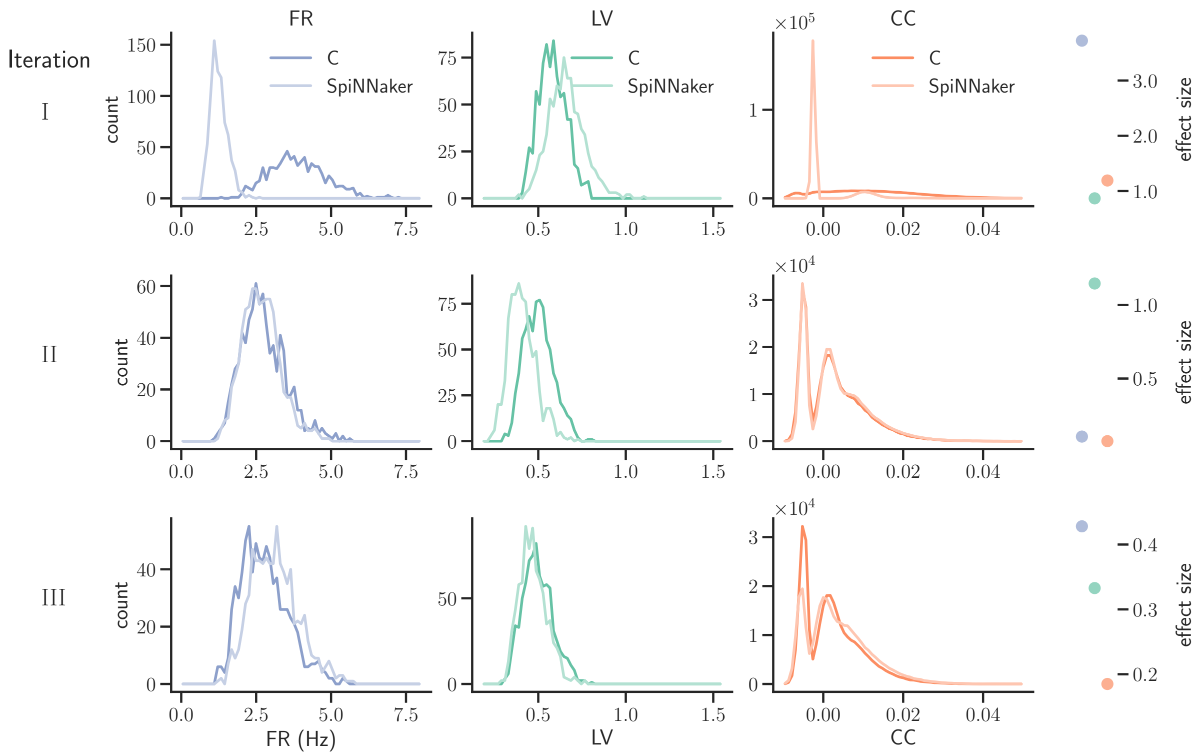

Figure S3. Characteristic measures computed from 60 s of network activity after 3 hours of simulation.

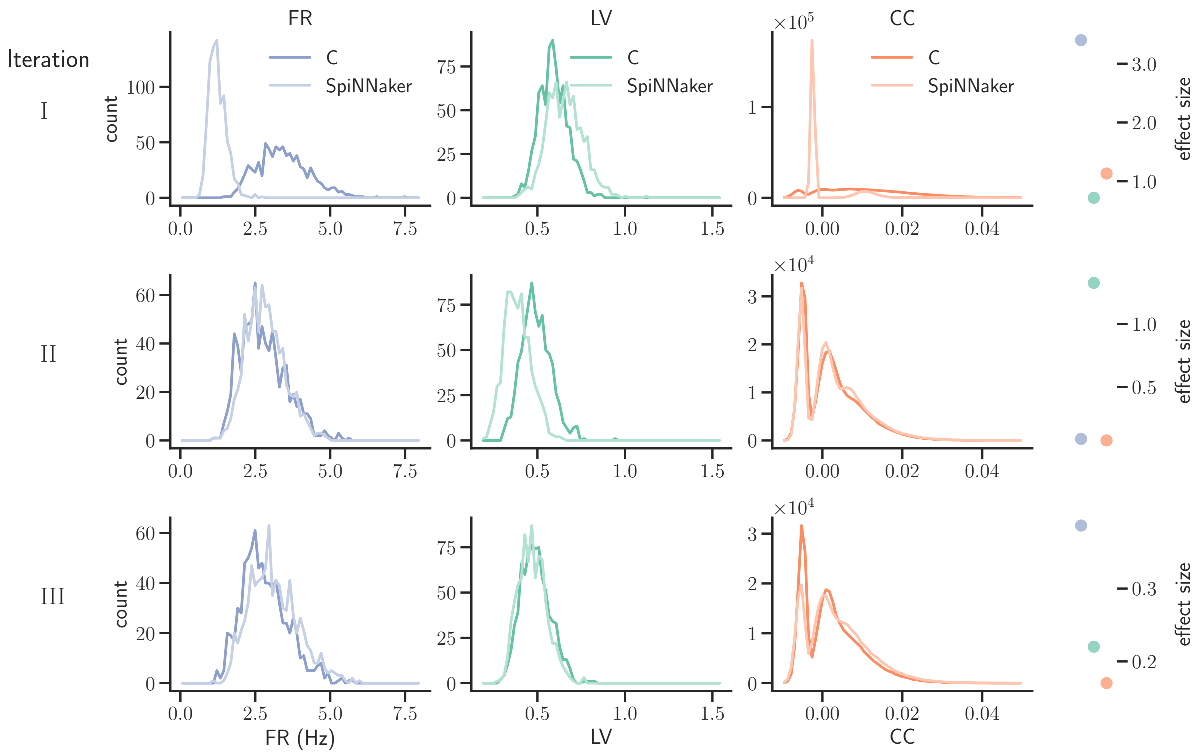

Figure S4. Characteristic measures computed from 60 s of network activity after 4 hours of simulation.

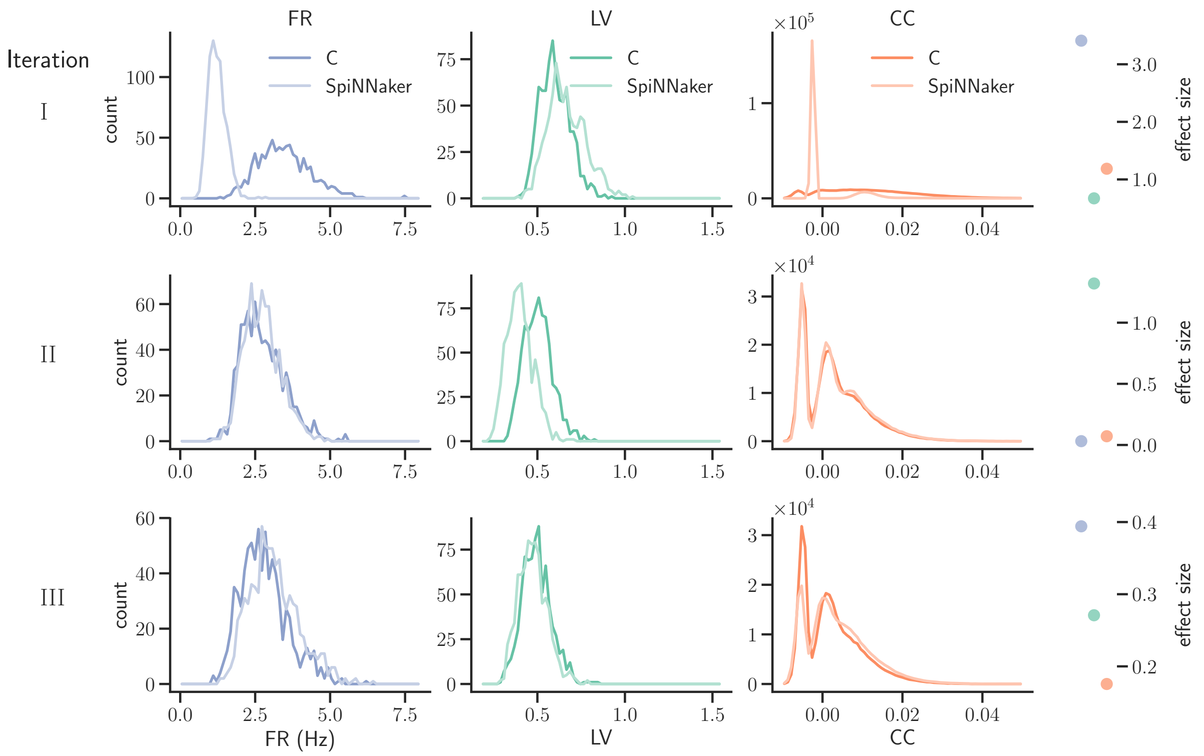

Figure S5. Characteristic measures computed from 60 s of network activity after 5 hours of simulation.
